# Intelligence

**Published:** 1829-09

**Authors:** 


### INTELLIGENCE.

### MONTHLY REPORT OF DISEASES.

Cholera has been very prevalent during the last three weeks, and in several instances, we understand, it has proved fatal. The cases which have fallen under our immediate observation have been mild, and easily controlled by medicine. Bilious diarrhœa, unaccompanied by any sickness, has also been general.

For the following account of a disease in some respects resembling cholera, which has very recently attacked many individuals in one family at Clapham, we are chiefly indebted to the Medical Gazette.

On Thursday, Aug. 14, a son of Mr. Day, a schoolmaster at Clapham, aged three years, having been previously in good health, was seized with violent vomiting and purging about six o'clock in the morning. At twelve convulsions came on, and he continued in them until seven o'clock on the following morning, when he expired.

All the other boys, thirty in number, between four and fourteen years of age, remained well the next day. This being Saturday, several of the pupils went home, leaving in the school twenty-two boys. Of these, twenty were attacked on Sunday morning with vomiting and purging of a very alarming character, attended with a degree of prostration which threatened many of them with immediate death. The appearance of the matters vomited was somewhat various in different individuals. In some instances it was tinged with green bile, and was of a subacid smell; but in the great majority of cases it was colourless and inodorous. The stools also varied in appearance, but they were for the most part pale, consisting of mucus and muco-purulent matter, slightly streaked with scarlet blood. In different boys, also, the pulse varied very much. In the early stages of collapse it was very frequent, but so feeble as to be scarcely perceptible. The skin was in most instances cold and clammy throughout. In a few cases it was for a short time hot, and the face was in these occasionally flushed. There was a low delirium in some advanced cases, with dilated pupils, but the sensorium was not affected in the greater number of them. There was no pain in the stomach or bowels, beyond the griping which preceded the stools. In a few of the cases there was slight tenderness, and some tension of the abdomen. Altogether, indeed, the disease came on very much like the tropical cholera, with a short obscure stage of excitement, immediately followed by a state of extreme collapse, which, under the use of stimulants, was succeeded, in the milder cases, by warmth, gentle moisture, and general reaction. In most of the cases there was convulsive action of the muscles; but it was rather a twitch or subsultus than cramp, and it was confined to the upper extremities.

On Sunday afternoon, Dr. Spurgin, and Messrs. Angus and Saunders, of Clapham, requested the co-operation of Dr. P. M. Latham, Dr. Chambers, and Mr. Pearson. Another of Mr. Day's sons was now evidently sinking; and a third, as well as several of the pupils, were in a state of dangerous collapse; others, again, although not out of peril, were rallying.

Every inquiry was made to ascertain if the children had eaten any thing which was likely to produce such symptoms; but upon this point no satisfactory information was obtained.

The following appearances were detected in the body of the child who was the first victim of the disease. On laying open the abdomen, the viscera appeared perfectly healthy, as far as external appearances went. Liver was healthy in size and colour. Gall-bladder somewhat distended with healthy bile. Peritoneum throughout pale, transparent, and perfectly free from any appearance of thickening. On laying open the small intestines, however, it was observed that the peyerian plexuses of mucous glands were enlarged in patches throughout the ileum, raising internally, without destroying the mucous membrane covering them, into condylomatous elevations. Lower down in the small intestines, a few of the glandulæ solitariæ were similarly affected; and, in the ascending colon and transverse arch, these latter glands seemed almost universally diseased, giving an appearance of pustulation, or rather tuberculation, to the whole interior of the bowel; the interstices of the tubercles here, as well as in the small intestine, being entirely free from vascularity. The mesenteric and mesocolic absorbent glands, in the neighbourhood of the parts most diseased, were congested and enlarged. The stomach thoracic viscera, and brain, were quite healthy.

The treatment which had been adopted, and which it was determined still to pursue, was to give stimulants, with opiates, to those who were sinking from exhaustion and spasm. In the few instances in which the head seemed, in the course of the reaction, to be affected, leeches were applied to the temples. Mustard poultices were applied to the abdomen. Clysters were given, and afterwards full doses of calomel and opium.

Early on Monday, another of Mr. Day's sons, æt. four years, died twenty-three hours after being attacked. His body was carefully examined a few hours after death, and exhibited the following appearances. The abdominal viscera, when first exposed, appeared perfectly healthy. The examination of the bowels was commenced with the ileum, in which the mucous glands, both aggregate and solitary, were found generally enlarged, and the mucous membrane covering them in many places ulcerated. The interior of the cæcum, colon, and rectum, however, exhibited no appearance of diseased mucous glands, although the membrane throughout was unitormly congested, pulpy, and very easily separable from the subjacent tissue. The examination was now pursued upwards from the ileum. The jejunum, at the lower part, was less diseased than the ileum, and, as it approached the duodenum, was more and more healthy. The duodenum, however, on being laid open, exhibited a pustulated appearance, depending on enlarged follicles, very similar to that of the colon in the former case. The mesenteric and mesocolic glands belonging to the diseased portions of bowel were enlarged, and more vascular than natural. The liver was quite healthy. The gall-bladder contained more than an ounce of perfectly healthy bile. It was remarkable that the contents of the bowels were nearly colourless, and had no feculent, or indeed any other peculiar odour. The stomach was perfectly healthy. Thoracic viscera also healthy. The ventricles of the brain were distended with about three ounces of serum, and the sinuses were somewhat more charged than usual with dark-coloured blood. The brain and its appendages were not otherwise diseased.

Most of the boys were removed by their friends on the 17th, many of them in a very alarming condition; but they all recovered in the course of the week.

It was afterwards ascertained that a very foul drain, or cesspool, the situation of which was not previously known, behind the house, was accidentally opened a day or two before the disease occurred. The contents of this receptacle were taken out, and thrown into a garden adjoining the play-ground, and separated from it only by a low and slight open paling. Whether the sulphureted hydrogen itself was the agent in producing this pestilence, or whether that gas was merely the vehicle of some more subtle miasma, is doubtful; but that the boys were freely exposed to this effluvia is quite certain; and that almost every one of those who had been in the play-ground was attacked by this disease, is also generally undoubted. It is remarkable that the younger boys were most severely affected, and that a man, who actually fell into the cesspool, escaped altogether.

---

*University of London.*—The medical classes commence on the 1st of October; the general classes on the 2d of November.

---

### MONTHLY LIST OF MEDICAL BOOKS.

### METEOROLOGICAL JOURNAL,

By Messrs. HARRIS and Co. Mathematical Instrument Makers, 50, High Holborn.

| July | Rain gauge. | Moon | Thermom. 9 A.M. | Thermom. MAX. | Thermom. MIN. | Barometer. 9 A.M. | Barometer. 10 P.M. | De Luc's Hygrom. 9 A.M. | De Luc's Hygrom. 10 P.M. | Winds. 9 A.M. | Winds. 10 P.M. | Atmospheric Variations. 9 a.m. | Atmospheric Variations. 2 p.m. | Atmospheric Variations. 10 p.m. |
|---|---|---|---|---|---|---|---|---|---|---|---|---|---|---|
| 20 | | | 61 | 70 | 50 | 29.90 | 29.99 | 48 | 48 | W | W | Fine | Fine | Fine |
| 21 | | | 65 | 75 | 59 | 30.10 | 30.10 | 48 | 47 | W | W | —— | —— | —— |
| 22 | | | 68 | 73 | 61 | .06 | .04 | 47 | 51 | W | W | Cloudy | —— | —— |
| 23 | | ☾ | 65 | 73 | 59 | .04 | 29.96 | 51 | 51 | W | W | Cloudy | —— | —— |
| 24 | | | 68 | 75 | 62 | 29.84 | .75 | 51 | 57 | SW | S | Fine | —— | Cloudy |
| 25 | | | 66 | 71 | 64 | .73 | .79 | 57 | 56 | SSE | SSW | Cloudy | —— | Fine |
| 26 | | | 68 | 73 | 49 | .86 | .91 | 55 | 54 | SW | SSW | Fine | Show'ry | —— |
| 27 | | | 61 | 63 | 53 | .96 | .97 | 54 | 50 | NNE | NNE | —— | Fine | —— |
| 28 | | | 60 | 62 | 54 | .93 | .89 | 48 | 48 | NNE | SW | —— | —— | —— |
| 29 | | | 61 | 68 | 52 | .55 | .58 | 50 | 52 | WSW | SW | Rain | —— | Cloudy |
| 30 | | ● | 59 | 63 | 52 | .63 | .66 | 52 | 57 | ESE | NE | Fine | Fine | Cloudy |
| 31 | .54 | | 61 | 65 | 52 | .82 | 30.00 | 55 | 53 | N | N | —— | —— | Fine |
| Aug. | | | | | | | | | | | | | | |
| 1 | | | 61 | 66 | 55 | 30.08 | .03 | 53 | 54 | N | N | —— | —— | —— |
| 2 | | | 61 | 71 | 53 | 30.00 | 30.00 | 54 | 55 | NW | NW | —— | —— | —— |
| 3 | | | 58 | 64 | 52 | 29.97 | 29.78 | 55 | 55 | WSW | WSW | —— | Rain | Rain |
| 4 | | | 59 | 65 | 53 | .68 | .63 | 52 | 52 | W | W | Cloudy | Cloudy | Cloudy |
| 5 | | | 60 | 66 | 59 | .77 | .92 | 51 | 54 | NW | NNW | Cloudy | Fine | Fine |
| 6 | | | 65 | 69 | 50 | .94 | .94 | 54 | 53 | NNW | NW | Fine | —— | —— |
| 7 | | ☽ | 61 | 68 | 59 | 30.03 | .05 | 53 | 53 | WNW | W | Cloudy | —— | —— |
| 8 | | | 66 | 74 | 59 | .02 | .01 | 51 | 50 | WSW | SW | Fine | —— | —— |
| 9 | | | 64 | 72 | 57 | 29.95 | .86 | 53 | 53 | SWva. | SWva. | —— | —— | Cloudy |
| 10 | | | 59 | 68 | 59 | .78 | .81 | 57 | 52 | WSW | W | Rain | —— | Fine |
| 11 | .105 | | 65 | 68 | 59 | .96 | 30.01 | 50 | 51 | W | SW | Fine | Cloudy | Fine |
| 12 | | | 68 | 72 | 61 | .98 | .88 | 51 | 51 | SW | SW | —— | Fine | —— |
| 13 | | | 70 | 74 | 60 | .64 | .55 | 50 | 53 | SW | SSW | Cloudy | —— | Cloudy |
| 14 | | ○ | 63 | 65 | 53 | .41 | .34 | 55 | 56 | S | NNE | Rain | Rain | Show'ry |
| 15 | | | 55 | 58 | 50 | .43 | .57 | 57 | 60 | NNW | NNW | Cloudy | Rain | Rain |
| 16 | .75 | | 57 | 62 | 47 | .84 | .96 | 58 | 55 | NW | SW | Fine | Cloudy | Fine |
| 17 | | | 56 | 66 | 55 | 30.03 | .97 | 52 | 55 | W | SW | Fine | Fine | —— |
| 18 | | | 57 | 64 | 57 | 29.67 | .54 | 55 | 56 | SSW | SW | Rain | Rain | Cloudy |
| 19 | .50 | | 62 | 64 | 54 | .40 | .33 | 50 | 56 | Wvar. | SW | Fine | Show'ry | Fine |

The quantity of Rain fallen in the month of July, was 3 inches and 44-100ths.

